# Microglia depletion/repopulation does not affect light-induced retinal degeneration in mice

**DOI:** 10.3389/fimmu.2023.1345382

**Published:** 2024-01-15

**Authors:** Nils Laudenberg, Urbanus Muthai Kinuthia, Thomas Langmann

**Affiliations:** ^1^ Laboratory for Experimental Immunology of the Eye, Department of Ophthalmology, Faculty of Medicine and University Hospital Cologne, University of Cologne, Cologne, Germany; ^2^ Center for Molecular Medicine Cologne (CMMC), University of Cologne, Cologne, Germany

**Keywords:** retina, microglia, PLX3397, degeneration, repopulation

## Abstract

Reactive microglia are a hallmark of age-related retinal degenerative diseases including age-related macular degeneration (AMD). These cells are capable of secreting neurotoxic substances that may aggravate inflammation that leads to loss of photoreceptors and impaired vision. Despite their role in driving detrimental inflammation, microglia also play supporting roles in the retina as they are a crucial cellular component of the regulatory innate immune system. In this study, we used the colony stimulating factor 1 receptor (CSF1R)-antagonist PLX3397 to investigate the effects of microglia depletion and repopulation in a mouse model of acute retinal degeneration that mimics some aspects of dry AMD. Our main goal was to investigate whether microglia depletion and repopulation affects the outcome of light-induced retinal degeneration. We found that microglia depletion effectively decreased the expression of several key pro-inflammatory factors but was unable to influence the extent of retinal degeneration as determined by optical coherence tomography (OCT) and histology. Interestingly, we found prominent cell debris accumulation in the outer retina under conditions of microglia depletion, presumably due to the lack of efficient phagocytosis that could not be compensated by the retinal pigment epithelium. Moreover, our *in vivo* experiments showed that renewal of retinal microglia by repopulation did also not prevent rapid microglia activation or preserve photoreceptor death under conditions of light damage. We conclude that microglia ablation strongly reduces the expression of pro-inflammatory factors but cannot prevent photoreceptor loss in the light-damage paradigm of retinal degeneration.

## Introduction

Age-related macular degeneration (AMD) is a common eye disorder in the Western world, leading to irreversible visual deficits in people above 55 years of age. Clinically, AMD is classified into different forms; an early form characterized by medium-sized drusen deposits in the macula and a late advanced stage that manifests as either an atrophic form (dry AMD) or exudative form (wet AMD) (1). Although the early stage is asymptomatic (1), drusen deposits accumulate in the subretinal space (SRS) during disease progression leading to destruction of retinal pigment epithelium, photoreceptor degeneration and expansion of atrophic area. The exudative form (wet AMD) is characterized by damage to Bruch’s membrane and angiogenic sprouting of choroidal vasculature and leakage into the retina. Several risk factors including age, smoking, oxidative stress, lipid abnormalities and genetic factors play key roles in the etiology of the disease (1, 2). Genetic variants in complement genes are among the genetic factors with a strong effect on AMD risk (3, 4). Activation of the retinal immune system and accumulation of mononuclear phagocytes in the subretinal space are a common hallmark for AMD pathology in humans (5). Of note, targeting the innate immune system has been recently proposed as a therapeutic option for the treatment of atrophic AMD (6).

Microglia are yolk sac-derived self-renewing immune sentinels of the CNS including the retina (7, 8) which play crucial roles in tissue homeostasis and pathological states (9, 10). Under homeostatic conditions, a mild parainflammation is resolved to maintain tissue integrity. However, latent parainflammation may become pronounced due to continued cellular stress leading to microgliosis and degeneration of the retina (11, 12). As reactive microglia are a common early pathological feature of AMD, these cells present an avenue for immunomodulatory therapies in the retina.

Within the CNS, microglia are distinguished from other glial cells by their origin, gene expression and function (13). The development, survival, proliferation and maintenance of microglia is dependent on signaling by colony stimulating factor 1 receptor (CSF1R), a class III receptor tyrosine kinase (14, 15). The signaling pathway is maintained by two CSF1R ligands; CSF1 and IL-34, which have distinct origin and primary amino acid sequence, but related tertiary structure (16). CSF1 is produced by astrocytes and oligodendrocytes whereas IL-34 is secreted by neurons. Mice deficient of CSF1R or either of the ligands (IL34 or CSF1) exhibit reduced microglia density and function within the CNS (17–19). Indeed, mice with spontaneous null mutation in the *Csf1* (*Csf1* ^op/op^ mice) have reduced populations of tissue-resident macrophages, a phenotype that could be rescued by transgenic local expression of CSF1 in these mice (20, 21). In adult mice, CNS tissue macrophages have been depleted with inhibitory anti-CSF1R antibody (22) or inhibitors of CSF1R tyrosine kinase activity (23).

The CSF1R inhibitors PLX3397 or PLX5622 are effective in crossing the blood-brain- and blood-retinal barrier and have been shown to deplete > 90% of microglia in the CNS (24). Therefore, oral administration of CSF1R inhibitors provides a systemic approach for non-invasive microglia depletion in adult mice. Although CSF1R inhibition seems adequate for CNS macrophage/microglia depletion, minor side effects on peripheral immune cells have been documented in some organs (25).

As self-renewing mononuclear phagocytes, microglia repopulate the mammalian CNS within one week after ablation with CSF1R antagonists or via genetic targeting in the *Cx3cr1* ^CreER^:iDTR system (26, 27). Despite some differences in the ablation efficiency by either CSF1R antagonists or diphtheria toxin, the depletion of microglia and repopulation has been shown to be neuroprotective and with capacity to induce a homeostatic pool of microglia beneficial for the neurons and vasculature (28).

Here we used a murine light damage paradigm that mimics some aspects of human atrophic AMD. Some important similarities between the light damage mouse model and human atrophic AMD include increased oxidative stress, photoreceptor and RPE degeneration as well as microglia activation in the outer retina (5, 29, 30). Additionally, the light damage model is very useful to study immune responses including chemokine signaling, macrophage recruitment and complement activation (31–33), which are involved in key pathogenic events of human atrophic AMD.

However, since mice lack a macula and have a lower density of cones in the central retina compared to the human macula, rodent models fail to recapitulate macula-specific characteristics of human atrophic AMD (34). Moreover, the etiology of human AMD involves both genetic and environmental factors such as aging, which is not properly recapitulated in the murine light damage model (35). Despite of these differences, the light damage model has the advantage of a specific and synchronized degeneration of photoreceptor cells in large numbers and light intensity can be titrated to determine the speed of the degeneration (30).

In the present study, we hypothesized that microglia depletion with PLX3397 could reduce neurotoxicity and limit retinal degeneration in a murine light damage paradigm. Furthermore, we speculated that repopulating microglia may possess homeostatic properties and could confer beneficial properties to the retinal microenvironment following acute inflammation. Overall, the purpose of both experimental approaches was to determine whether temporary microglia depletion could limit the extent of retinal degeneration whether before or after light exposure.

## Materials and methods

### Experimental animals

All mouse studies were done according to the standards set by the local government committee (Landesamt für Natur, Umwelt und Verbraucherschutz Nordrhein-Westfalen). Animals were housed in individually ventilated cages (M 500, Tecniplast^®^ Greenline) with a maximum of 5 animals per cage under specific pathogen free (SPF) conditions. The light was tuned to a 12h/12h light/dark cycle while the temperature and relative humidity were maintained at 22 ± 2°C and 45-65%, respectively. The animals were provided with access to acidified water and standard rodent diet (Altromin 1314) free of phytoestrogen *ad libitum*.

Male and female mice aged 8 to 12 weeks were used in the experiments. Heterozygous CX3CR-1^GFP/-^ reporter mice, were kindly provided by Dr. Deniz Hos. These knock-in animals carry an enhanced green fluorescent protein (EGFP) sequence, inserted before the first 390 base pairs of exon 2 of the *chemokine (C-X3-C motif) receptor 1 (Cx3cr1)* gene. As a result, EGFP is expressed by monocytes, dendritic cells, NK cells, brain microglia, and retinal microglia. The mice were back-crossed with the BALB/cJ strain to generate mice carrying the light-sensitive RPE-specific protein (65kDa) (RPE65) Leu450 variation.

### PLX3397 chow

PLX3397 hydrochloride, a CSF1R inhibitor, was purchased from MedChemExpress and mixed with standard rodent diet at final concentrations of 150 ppm and 1,200 ppm, respectively. The mice received the PLX3397 diet for a maximum of 14 days depending on the experimental setup. Control mice were fed standard rodent diet provided by Altromin. The concentration of PLX3397 in the retina, liver and brain samples from experimental mice was determined by liquid chromatography - mass spectrometry (LC-MS).

### Light-damage paradigm and microglia depletion

Mice were housed for 16 hours in the dark before exposure to light. The pupils were dilated with 2.5% phenylephrine and 0.5% tropicamide and the mice were placed in separate cages with reflective aluminum foil coating, followed by a 1 hour exposure to bright white light with intensities of 5,000 lux or 15,000 lux. Following exposure to light, the mice were kept under a 12h/12h light/dark cycle.

In the first experimental setup we examined the therapeutic effect of microglia depletion in a mouse model of acute retinal degeneration. For these experiments, we established four treatment groups. Group 1 and 2 were not exposed to light with group 1 receiving the standard rodent diet whereas group 2 received the PLX3397 diet. In contrast, group 3 and 4 were exposed to 15,000 lux of white light for one hour to induce retinal degeneration. In order to ensure that microglia were depleted prior to the initiation of retinal degeneration, group four received the PLX3397 diet starting 7 days before the light exposure and lasted until the time of analysis, while group three received a control diet. Mice were sacrificed 4 days following light exposure and the eyes were collected and processed for further examination.

In the second experimental setup we sought to investigate the effects of microglia depletion 14 days after the onset of light-induced retinal degeneration. Consequently, all four groups were exposed to 5,000 lux of white light to exhibit a slow but progressive degeneration. The effects of both early and late depletion of microglia during retinal degeneration were examined.

### *In vivo* imaging

In order to anesthetize the mice for imaging, ketamine (Ketavet, 100 mg/kg) and xylazine (Bayer, 2% Rompun, 5 mg/kg) were injected intraperitoneally. To dilate the pupils, 2.5% phenylephrine and 0.5% tropicamide eye drops were applied topically. Spectral-domain optical coherence tomography (SD-OCT) and BluePeak laser autofluorescence (BAF) were used with the Spectralis™ HRA/OCT device (Heidelberg) to measure retinal thickness and fundus autofluorescence, respectively. Retinal thickness heatmaps were generated using the HEYEX software. The average values for the four segments within the diameters of 3 mm (central retina) and 6 mm (peripheral retina) from the optic nerve head, were used to compute the thickness for each eye.

### Immunohistochemistry

Enucleated eyeballs were fixed in 4% paraformaldehyde (PFA) at room temperature (RT) for two hours. Following dissection of the retina from RPE, retinas were permeabilized, and unspecific antigen binding sites were blocked with Perm/Block buffer (5% goat serum, 0.2% BSA and 0.3% Triton X-100 in PBS) at 4°C overnight. Subsequently, retinal whole mounts were incubated in the blocking solution with the primary antibodies presented in **Table 1** below.

**Table 1:** List of antibodies used for immunohistochemistry.

Antibodies	Species	Dilution	Manufacturer, Cat. No
**anti-GFAP**	Rabbit, polyclonal	1:500	Sigma-Aldrich; G9269
**anti-cone arrestin**	Rabbit, polyclonal	1:500	Sigma-Aldrich; 32160702
**Alexa Fluor^®^ 594**	Donkey anti-rabbit IgG	1:800	Invitrogen; A11007
**Alexa Fluor^®^ 647**	Donkey anti-rabbit IgG	1:800	Invitrogen; A-31573
**Alexa Fluor^®^ 350**	Donkey anti-rabbit IgG	1:800	Thermo Fisher; A10039
**Alexa Fluor^®^ 594**	Goat anti-mouse IgG	1:800	Thermo Fisher; A-11005

After several washing steps in PBST-X (0.3% Triton X-100 in PBS), the samples were incubated for 2 hours at RT with the secondary antibodies indicated in **Table 1**, diluted in PBST-X. Finally, retinal flat mounts were embedded with Vectashield^®^ HardSet™ or DAKO mounting medium, placed on microscope slides and allowed to dry before examination under the microscope.

For immunohistochemical analysis of retinal sections, fixed eyes were incubated with an increasing concentration of sucrose (10%, 20% and 30%) for dehydration. Thereafter, eyes were embedded in Optimal Cutting Temperature (O.C.T.™) medium in cryomolds and placed on dry ice. Retinal sections with a thickness of 10 µm were cut using a cryostat (Leica, CM3050S) and stored at -20°C until further processing. Frozen slides were thawed and rehydrated in PBS for 10 minutes before blocking unspecific antigens with BLOTTO buffer (1% Non-fat dried milk powder and 0.3% Triton X-100 in PBS) at RT for 30 minutes. Subsequently, sections were incubated in an antibody solution (2% BSA and 0.1% Triton X-100 in PBS) with the primary antibodies (**Table 1**) overnight at 4°C. After several washing steps in PBS, sections were incubated in PBS with the secondary antibodies (**Table 1**) for one hour at RT. Following the final washing steps, the sections were embedded in mounting medium with DAPI and covered with a glass cover slip to dry before microscopy. Images of all samples were acquired with a Zeiss Imager.M2 equipped with an ApoTome.2.

### TUNEL staining

The *in situ* cell death detection kit RED (Roche) was used in accordance with the manufacturer’s instructions to detect and quantify retinal cell death on cryosections using terminal deoxynucleotidyl transferase dUTP nick end labeling (TUNEL).

### RNA isolation, reverse transcription and quantitative real-time PCR

Total RNA was extracted from mouse retinas using the RNeasy^®^ Micro Plus kit (Qiagen) following the manufacturer’s instructions. RNA integrity and quantity were assessed and quantified spectrophotometrically with a NanoDrop 2000 (Thermo Scientific) First-strand cDNA was synthesis was carried out with the RevertAid H Minus First-strand cDNA Synthesis kit (Thermo Scientific) according to the manufacturer’s guidelines.

Quantitative real-time PCR was performed in the LightCycler^®^ 480 II (Roche) with SYBR^®^ Green (Takyon No Rox SYBR Master Mix dTTP blue, Eurogentec) detection to determine the mRNA transcript levels of selected markers. The primer sequences are provided in **Table 2**. Transcript measurements were performed in technical duplicates and *Atp5b* expression was used as a reference gene and the results were presented as relative mRNA expression using the delta delta CT threshold method for relative quantification.

**Table 2:** Primer sequences and NM accession numbers for qRT-PCR targets.

Gene	Forward primer (5’-3’)	Reverse primer (5’-3’)	NM accession number
***Atp5b***	ggcacaatggaggaaagg	tcagcaggcacatagatagcc	NM_016774.3
***Aif-1***	ggatttgcagggaggaaaag	tgggatcatcgaggaattg	NM_019467.4
***Il-1β***	tgtaatgaaagacggcacacc	tcttctttgggtattgcttgg	NM_008361.4
***Il-6***	gctaccaaactggatataatcagga	ccaggtagctatggtactccagaa	NM_001314054.1
***Tspo***	ggaacaaccagcgactgc	gtacaaagtaggctcccatgaa	NM_009775.4
***Tnfα***	ctgtagcccacgtcgtagc	ttgagatccatgccgttg	NM_001278601.1
***Ccl2***	catccacgtgttggctca	gatcatcttgctggtgaatgagt	NM_011333.3

### Image analysis

The public and fully automated analytical tool MotiQ (version 3.1.1), a Java plugin for ImageJ (FIJI), was used to perform the morphometric analysis of microglia in retinal flat mounts. Analyses were performed on two-dimensional images with mean intensity projection (MIP) and 6 morphological parameters were quantified. The overall complexity of the cells is described with an arbitrary value known as the ramification index which is the ratio of cell surface area to cell volume. The cells with a high ramification index have long, branched processes and occupy a large area.

The particle analyzer tool from FIJI was used to count the number of cone-arrestin-positive cells in retinal flat mounts. The percentage of TUNEL-positive cells in the ONL was calculated to assess the cell death rate. The total number of both, ONL and TUNEL-positive cells were counted with FIJI’s multi-pointer tool. To analyze microglia migration, CX3CR1-GFP expressing cells were counted in the ONL, OPL, INL, and IPL using the multi-pointer tool.

### Statistical analysis

Throughout the study, all data were analyzed and plotted with GraphPad Prism (version 8.4.3). The *in vivo* data were subjected to a normality test prior to statistical analysis. A two-way ANOVA was used followed by Tukey’s multiple comparison test (*p < 0.05, **p < 0.01, ***p ≤ 0.001). n-numbers and data points represent one retina from at least three independent animal experiments. Data is presented as the mean ± standard deviation along with error bars.

## Results

### Depletion of microglia does not rescue photoreceptors from light-induced degeneration

**Figure 1:**
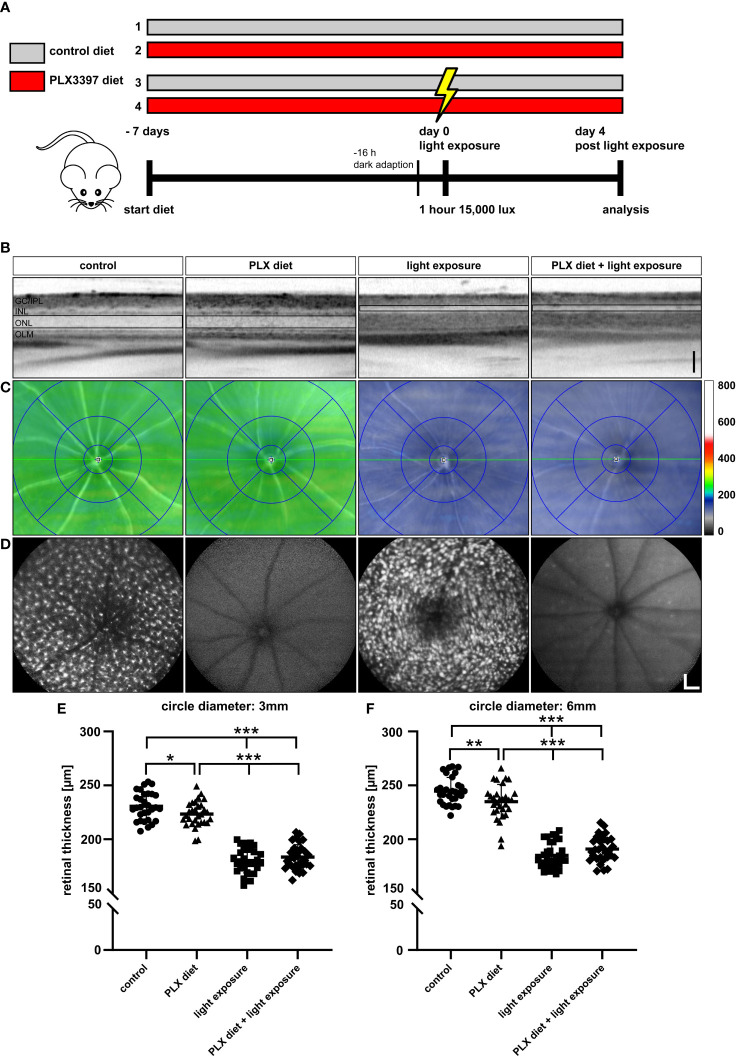
Microglia depletion in the light damage model of retinal degeneration. **(A)** PLX3397 diet was administered to *CX3CR-1^GFP/-^* reporter mice 7 days before exposure to white light with an intensity of 15,000 lux for 1 hour. Two control groups were not exposed to white light with one group receiving the PLX3397 diet and the other the standard rodent diet as illustrated in the experimental design. **(B)** SD-OCT scans show reflectance in the ONL, which was altered in light-exposed mice fed with either standard rodent diet or PLX3397 chow. **(C)** SD-OCT heatmaps of the indicated conditions. **(D)** Representative blue autofluorescence images showing the efficiency of PLX3397 in depleting microglia cells in the retina. Measurements of total retinal thickness within 3 mm **(E)** and 6 mm **(F)** from the optic nerve head shows that microglia depletion with PLX3397 does not prevent loss of photoreceptors **(E)**. PLX3397 diet triggered thinning of the retina without exposure to light. Data are presented as mean ± SD. *p < 0.05, **p < 0.01, ***p ≤ 0.001, n = 30 eyes. Black scale bar = 100 μm; white scale bar = 200 μm. GC/IPL; ganglion cell/inner plexiform layer, INL; inner nuclear layer, ONL; outer nuclear layer, OLM; outer limiting membrane.

Microglia activation is a hallmark of retinal degenerative diseases and we have previously demonstrated that pharmacological or genetic modulation of microglia-related genes including TSPO and galectin-3 can delay retinal degeneration (36, 37). As retinal microglia are dependent on CSF1R signaling for survival and proliferation, we tested here, whether microglia depletion could also be beneficial in a model of light-induced retinal degeneration. For this purpose, *CX3CR-1^GFP/-^* reporter mice were fed control diet or a diet supplemented with a specific CSF1R inhibitor, PLX3397 (**Figure 1A**). As a pilot study, we quantified the amount of PLX3397 in retinal tissue by LC-MS following 1 week feeding of mice with either the standard rodent diet,150 ppm PLX3397-supplemented chow, or 1200 ppm PLX3397 containing diet. These results confirmed that PLX3397 dose-dependently reached the retina (**Supplementary Figures 1A-C**). The *CX3CR-1^GFP/-^* reporter mice express GFP under the *Cx3cr1* promoter which enables *in vivo* imaging of resident microglia cells. Therefore, we could use blue autofluorescence (BAF) imaging to assess the potency of PLX3397 diet in depleting microglia. Mice fed the 1200 ppm PLX3397 containing diet showed retinal microglia depletion, whereas the diet containing 150 ppm was not effective (**Supplementary Figures 1D-F**).

We then investigated the effect of continuous PLX3397 treatment on photoreceptor degeneration, 4 days after exposure to 15,000 lux white light. The SD-OCT scans showed that light exposure triggered significant photoreceptor loss and thinning of the ONL (**Figure 1B**). PLX3397 diet had no significant effect on the extent of photoreceptor loss when compared to the control diet and light-exposed retinas (**Figure 1B**), as shown in SD-OCT heatmaps (**Figure 1C**). The BAF images showed that PLX3397 was highly effective in depleting microglia in both light exposed and control retinas (**Figure 1D**). BAF imaging also demonstrated that the light exposure activated microglia, which was manifested by a substantially larger and amoeboid morphology in the control diet group (**Figure 1D**). Quantification of total retinal thickness revealed that PLX3397 diet had no significant rescue effect on photoreceptors within the central or peripheral retina (**Figures 1E, F**). In contrast, PLX3397-fed animals showed a slightly thinner retina in non-light exposed retinas (**Figure 1E**).

### Efficient depletion of amoeboid microglia under light damage conditions

**Figure 2:**
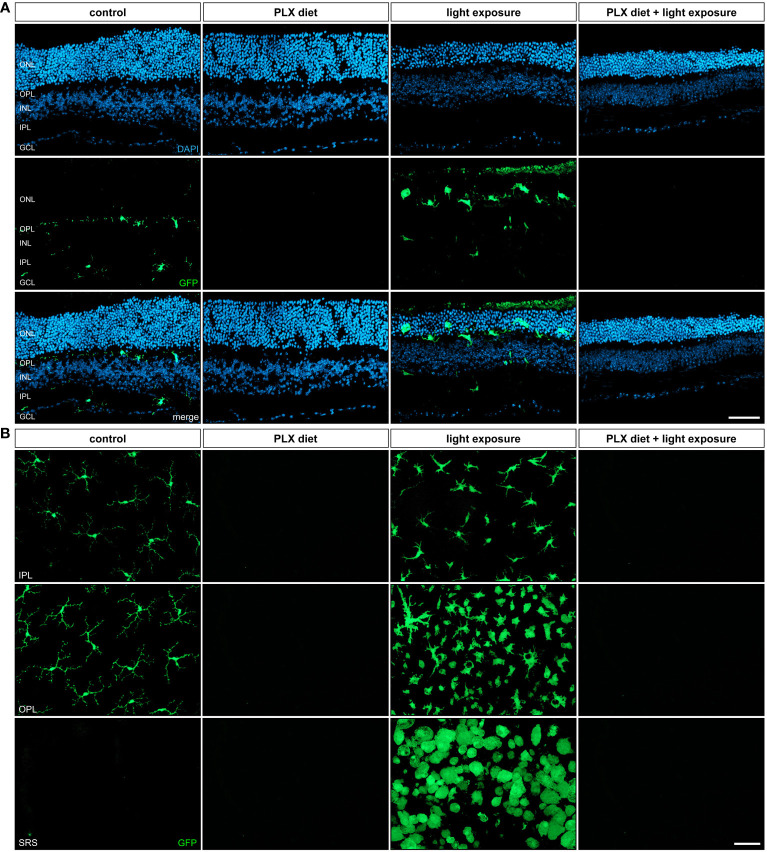
Retinal microglia phenotypes in light damage conditions. GFP-autofluorescent cells were analyzed in **(A)** cryosections and **(B)** flat mounts by microscopy for the conditions described in **Figure 1**. GFP-positive cells were present in control conditions and light-damage conditions and absent in retinas from mice receiving PLX3397 diet. **(A)** Microglia migration into the nuclear layers was triggered by light damage and **(B)** cells in the subretinal space (SRS) showed a bloated amoeboid morphology **(A)**. Scale bar = 50 µm.

A key feature of microglia activation is the migration from the plexiform layers to the site of degeneration, including the outer nuclear layer in photoreceptor damage. Our analyses of retinal sections showed that PLX3397 was effective in depleting GFP expressing cells in the retina with and without exposure to light (**Figure 2A**), which is in good accordance with the previously performed *in vivo* imaging. Exposure to light triggered amoeboid microglia morphology and migration to the degenerating outer nuclear layer (**Figure 2A**). We could not observe GFP+ cells migrating into the nuclear layers of either light-exposed mice or non-light exposed animals following treatment with PLX3397 (**Figure 2A**).

The investigation of retinal flat mounts further confirmed the largely amoeboid shape of microglia in light-exposed retinas (**Figure 2B**). Microglia states are complex and no longer fit into a dichotomic classification of good vs bad cells (38). However, it is well accepted that the activation of microglia causes an amoeboid morphology. Upon exposure to light, we observed an accumulation of amoeboid microglia cells in the plexiform layers and subretinal space, which is accordance with previous studies (39). Conversely, no microglia were detected in any of the retinal layers after PLX3397 treatment independent of the light exposure conditions.

### Significant blocking effects of PLX3397 on pro-inflammatory factor gene induction during light damage

**Figure 3:**
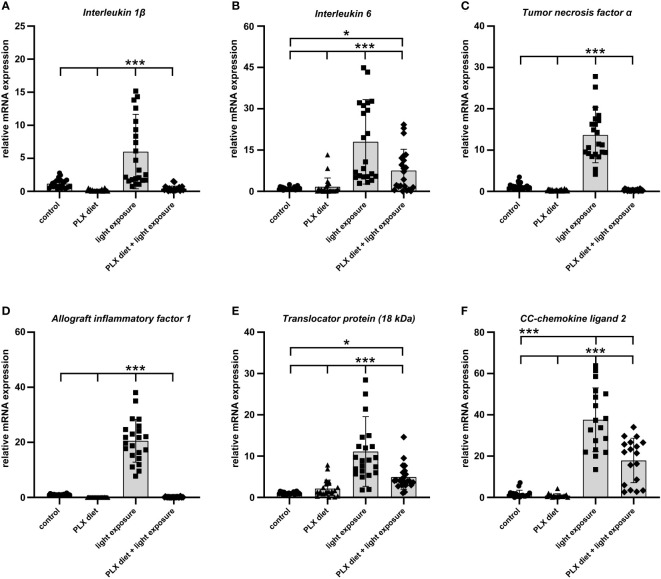
PLX3397 treatment limits the expression of pro-inflammatory factors in the retina. **(A–E)** qRT-PCR analyses showed that exposure to light induced upregulation of pro-inflammatory genes (*Il-1β, Il-6* and *Tnf-α*) and markers of microglia activation (*Aif-1* and *Tspo).* The induction of these genes was significantly attenuated in PLX3397 treated retinas. Although PLX3397-treated retinas showed lower transcript levels of *Il-6, Tspo* and *Ccl2* **(F)** compared to light-exposed retinas, their levels remained significantly higher than in control retinas. Data are presented as mean ± SD. *p < 0.05, **p < 0.01, ***p ≤ 0.001, n = 22 retinas.

Since reactive microglia commonly express high levels of pro-inflammatory factors in models of retinal degeneration (40, 41), we analyzed whether microglia depletion could prevent the secretion of inflammatory markers in acute retinal light damage. Relative quantification of inflammatory marker transcripts by qRT-PCR revealed that *Il-1β, Il-6, TNF-α, Aif-1, Tspo* and *Ccl2* were significantly upregulated with exposure to light when compared to control retinas (**Figure 3**). The light-damage induced expression of all these microglia transcripts was significantly reduced and for some genes even completely prevented in PLX3397 treated animals (**Figure 3**).

### Microglia depletion increases apoptotic cell death but does not affect cone cell numbers

**Figure 4:**
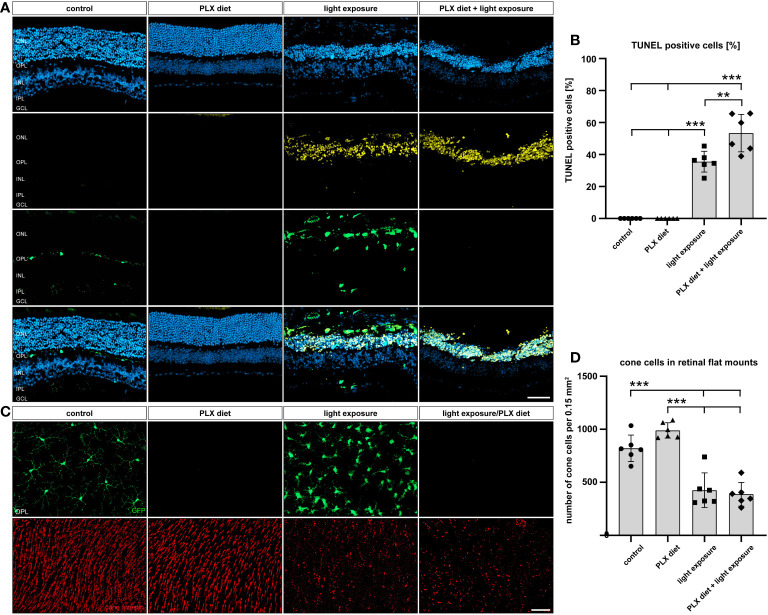
Effect of microglia depletion on cell death. **(A, B)** TUNEL stainings were performed on retinal sections from *CX3CR-1^GFP/-^* reporter mice for the conditions described in **Figure 1**. No TUNEL-positive cells were detected in retinas from mice that received PLX3397 diet or control mice without light exposure. In light-damaged retinas, 38% TUNEL^+^ cells were detected in the ONL, whereas the number of TUNEL^+^ cells significantly increased to 57% in the PLX337 treated group. **(C, D)** GFP-imaging and cone arrestin stainings were carried out to examine cone photoreceptor cell degeneration. A significant decrease in the number of cones was counted in light-damage conditions, which was not influenced by microglia depletion. Data are presented as mean ± SD. *p < 0.05, **p < 0.01, ***p ≤ 0.001, n = 6 retinas. Scale bar = 50 μm.

Phagocytic removal of apoptotic cell debris is a key feature of microglia in the CNS (42). We therefore analyzed the effect of microglia depletion on apoptotic cell death by TUNEL stainings. No apoptotic cells were observed in control and PLX3397-treated retinas in the absence of light damage (**Figures 4A, B**). A high number of TUNEL-positive cells was seen in light-exposed conditions, whereas microglia depletion even caused a significantly higher number of dead cells (57% of TUNEL+ cells versus 38% of TUNEL^+^ cells) in the outer retina 4 days after light-damage. Obviously, microglia depletion under degenerative conditions leads to failed phagocytosis and accumulation of cell debris in the outer retina.

We next counted the number of cone cells in flat mounts as another parameter to quantify retinal cell death (**Figures 4C, D**). A strong reduction in cone photoreceptor staining was noted after light exposure, but the PLX3397 diet did not result in additional effects. Thus, the light-induced decline in cone photoreceptor density was not influenced by microglia depletion.

### Repopulating microglia are rapidly activated but fail to confer a rescue effect on retinal degeneration

**Figure 5:**
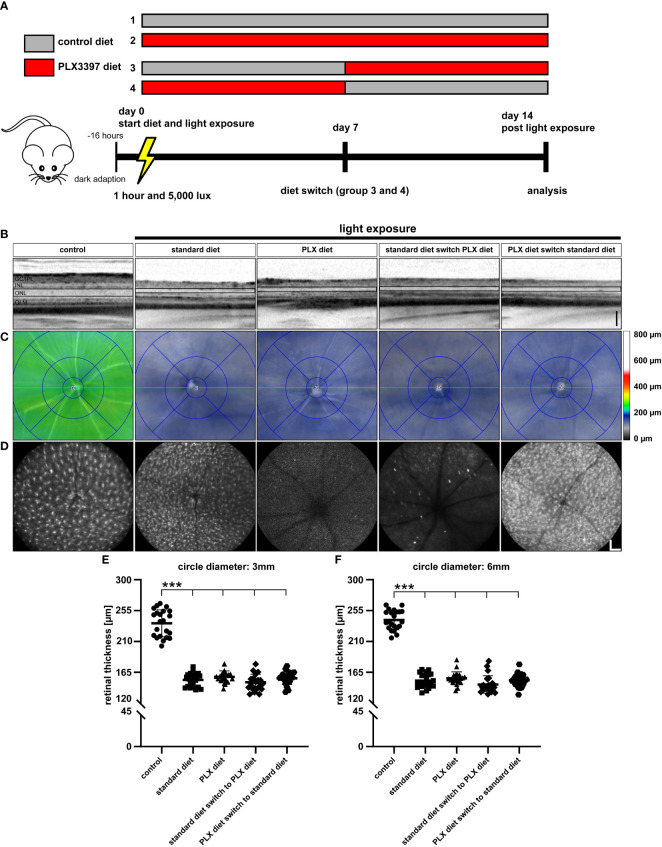
Microglia repopulation in the light damage model of retinal degeneration. **(A)** Experimental design for microglia depletion and repopulation at lower light intensity. *CX3CR-1^GFP/-^* reporter mice were dark adapted and then exposed to 5,000 lux for one hour. Group 1 received standard rodent diet for 14 days after exposure to light, while group 2 received the PLX3397 diet. Group 3 received standard diet from day 0 to day 6, which was replaced with PLX3397 diet from day 7 to day 14. Group 4 started with PLX3397 diet for seven days and was then switched to standard diet. **(B)** SD-OCT scans showing reflectance in the ONL, which was altered in light-exposed mice fed with either standard rodent diet or PLX3397 chow. **(C)** SD-OCT heatmaps revealed extensive ONL thinning under all conditions of light exposure compared to control mice without light exposure. **(D)** Blue autofluorescence imaging revealed efficient microglia depletion in groups 2 and 3 and a large number of repopulated cells in group 4. Quantification of retinal thickness within **(E)** central and **(F)** peripheral regions revealed no effects of microglia depletion or repopulation on retinal degeneration. Data are presented as mean ± SD. *p < 0.05, **p < 0.01, ***p ≤ 0.001, n = 22 eyes. Black scale bar = 100 μm, white scale bar = 200 μm. GC/IPL; ganglion cell/inner plexiform layer, INL; inner nuclear layer, ONL; outer nuclear layer, OLM; outer limiting membrane.

In the next set of experiments, we studied the effects of microglia depletion after exposure with lower light intensity (5,000 lux) for a longer period (**Figure 5A**). The first two experimental mouse groups received standard or PLX3397 diet for two weeks, respectively. The diets in groups three and four were switched on day 7 to examine the effects of early and late microglia depletion, respectively, followed by analysis at day 14. SD-OCT images of the retinal layers revealed a significant thinning after light exposure irrespective of the administered diet (**Figures 5B, C**). *In vivo* imaging of GFP-expressing cells revealed that microglia depletion was only present in animals that received PLX3397 diet throughout the experimental period and in diet switch group 3 (switch from standard to PLX3397 chow) (**Figure 5D**). Further quantitative analyses then showed that retinal thickness significantly decreased in all groups exposed to light irrespective of the diet (**Figures 5E, F**).

**Figure 6:**
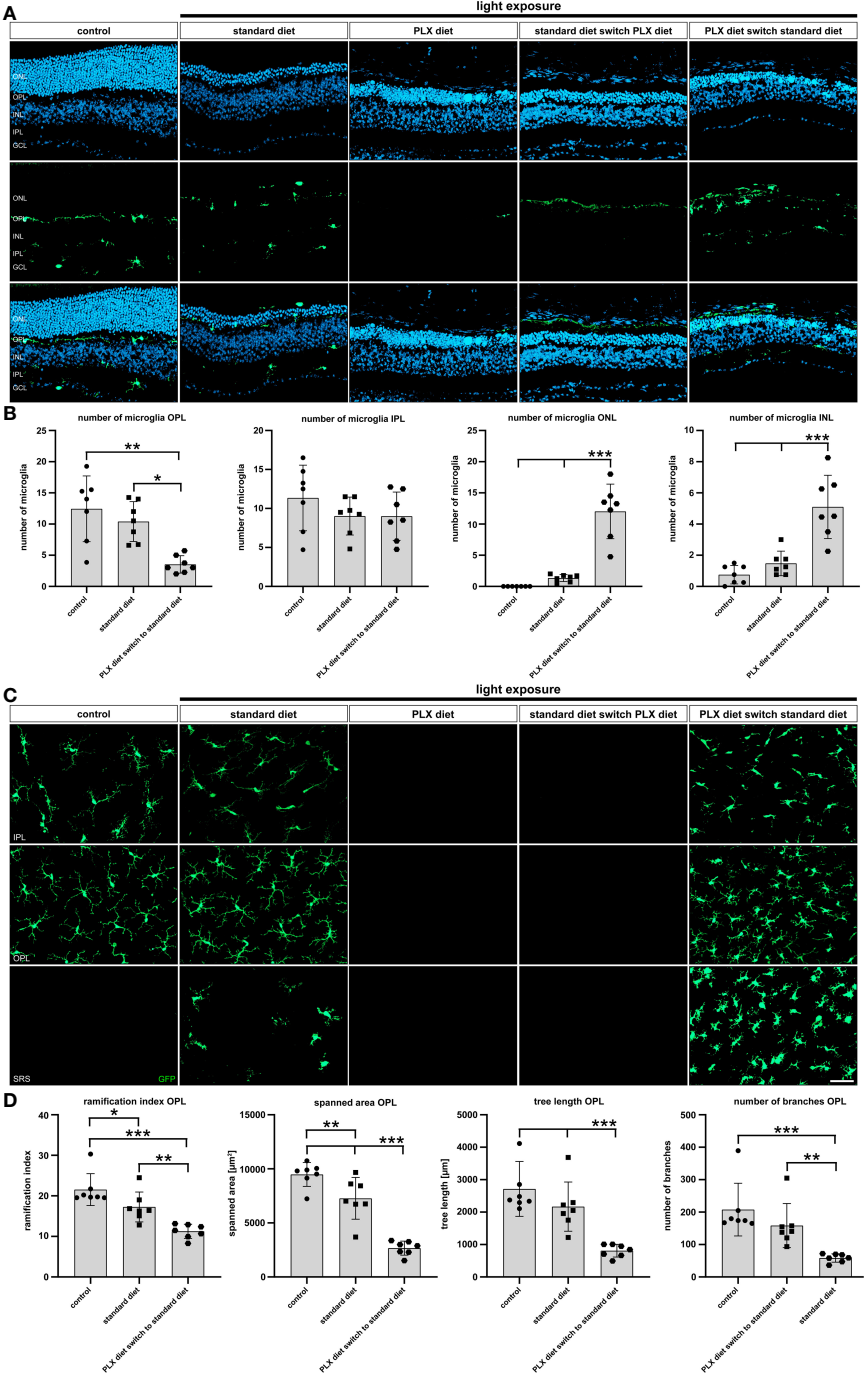
Effects of depletion and repopulation on microglia location and morphology. GFP-autofluorescent cells were analyzed in **(A, B)** cryosections and **(C, D)** flat mounts by microscopy for the conditions described in **Figure 5**. GFP-positive cells were present in control conditions and light-damage conditions under standard diet and PLX3397 diet switch to standard diet. GFP-positive cells were absent in conditions of PLX3397 depletion and PLX3397 diet switch to standard diet. **(A–C)** Following repopulation, a significant increase in the number of microglia was observed and these cells were mainly present in the outer nuclear layer. **(C, D)** Morphometric analysis with MotiQ showed that repopulating microglia predominantly displayed an amoeboid morphology as indicated by their lower ramification index, spanned area, tree length and branch density compared to controls. Data are presented as mean ± SD. *p < 0.05, **p < 0.01, ***p ≤ 0.001, n = 7 retinas. Scale bar = 50 μm. GC; ganglion cell layer, IPL; inner plexiform layer, INL; inner nuclear layer, OPL; outer plexiform layer, ONL; outer nuclear layer.

Next, the presence and location of microglia in retinal sections was investigated 14 days after low intensity light exposure. As expected, microglia-specific GFP signals were detected in control retinas and retinas of light-damaged mice under standard diet (**Figure 6A**). Continuous PLX3397 treatment and diet switch from standard to PLX3397 chow both showed absence of GFP-positive cells after 14 days (**Figure 6A**). Early microglia depletion was associated with a significantly lower number of newly repopulated microglia in the outer plexiform layer and higher numbers in both nuclear layers (**Figure 6B**). There were no differences in the number of microglia in the inner plexiform layer in any of the experimental conditions (data not shown).

To better describe the morphology of microglia in the different experimental conditions, we conducted morphometric analyses on retinal flat mounts 14 days after light exposure (**Figures 6C, D**). As already seen in the analyses of retinal sections, GFP-signals were absent in retinas of mice with light damage and complete as well as late microglia depletion. Highly ramified cells were present in the plexiform layers of control animals and light-damaged animals, whereas the latter also displayed amoeboid cells in the subretinal space (**Figure 6C**). In contrast, repopulated microglia showed a less ramified and more round appearance (**Figure 6C**) in the plexiform layers and subretinal space, that could be well corroborated by quantitative parameters including ramification index, spanned area, tree length and the number of branches (**Figure 6D**). We conclude that microglia that repopulate in the light-exposed condition are immediately triggered by the degenerating retinal environment to adopt an activated immune cell phenotype.

**Figure 7:**
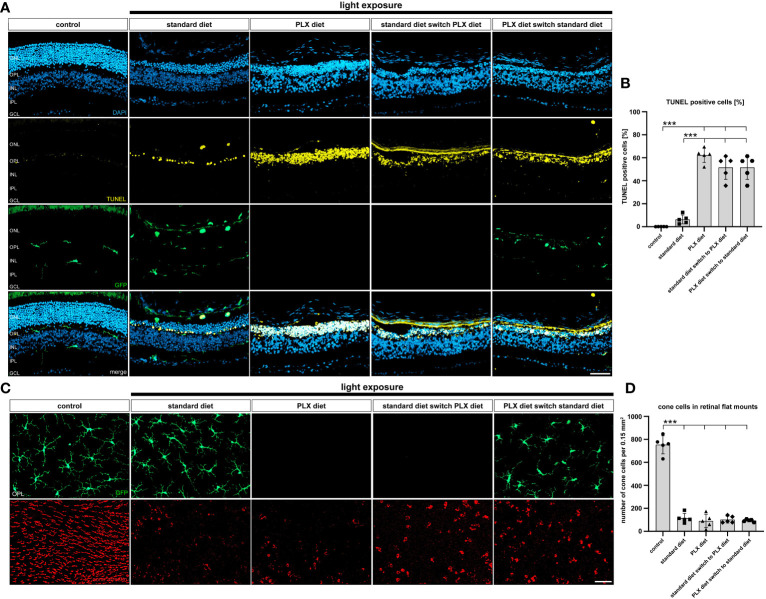
Effect of microglia depletion and repopulation on photoreceptor cell death. *CX3CR-1^GFP/-^* reporter mice received PLX3397 diet as indicated and 5,000 lux light exposure for one hour. **(A, B)** TUNEL stainings of cryosections and **(C, D)** cone-arrestin stainings of flat mounts were carried out 14 days after light exposure. **(B)** There was a significant increase in TUNEL-positive cells within the ONL in all animals that received PLX3397 diet. **(C, D)** Analysis of cone-arrestin staining showed that light exposure triggered a significant loss of cone photoreceptors independent of the diet. Data are presented as mean ± SD. *p < 0.05, **p ≤ 0.01, ***p ≤ 0.001, n = 5 retinas. Scale bar = 50 μm. GC; ganglion cell layer, IPL; inner plexiform layer, INL; inner nuclear layer, OPL; outer plexiform layer, ONL; outer nuclear layer.

We then performed TUNEL staining to evaluate the number of dying cells in the ONL after low light exposure. Our findings showed that the number of TUNEL-positive cells was significantly increased in all conditions of microglia depletion regardless of whether microglia were depleted before or after onset of retinal degeneration (**Figure 7A**). Of note, there was a significantly lower number of apoptotic cells within the outer retina of light-exposed mice fed standard diet, where microglia were present throughout the process of retinal degeneration (**Figure 7B**). As previously seen for the stronger light-damage conditions, microglia depletion under slower degenerative conditions also leads to higher accumulation of cell debris in the outer retina. The effect of microglia repopulation on cone photoreceptor survival was then examined 14 days after low light exposure. Exposure to 5000 lux white light caused a significant loss of cone photoreceptors, which was not significantly influences by depletion or repopulation of microglia (**Figure 7C, D**).

### Microglia repopulation is associated with a strong pro-inflammatory response in the degenerating retina

**Figure 8:**
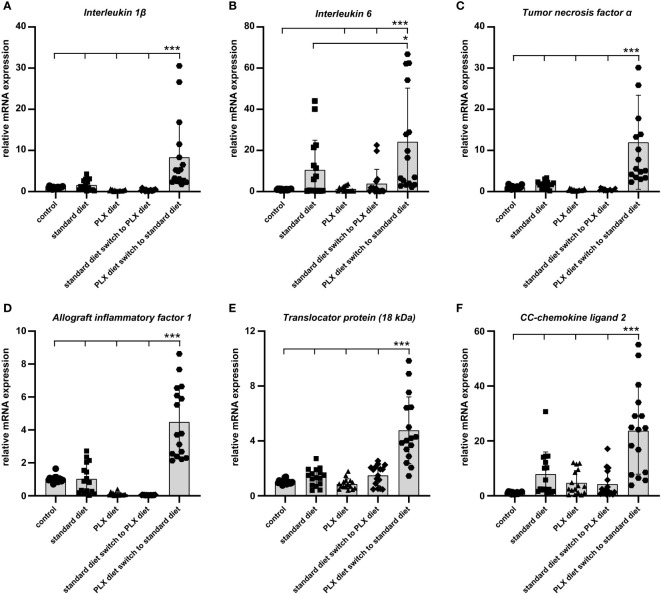
Effect of microglia depletion and repopulation on pro-inflammatory marker expression. *CX3CR-1^GFP/-^* reporter mice received PLX3397 diet as indicated and 5,000 lux light exposure for one hour. **(A–F)** qRT-PCR was used to quantify levels of pro-inflammatory cytokines in whole retina tissue14 days after light exposure. In newly repopulated microglia, the expression of pro-inflammatory markers significantly increased when compared to the light exposure group under standard diet. Data are presented as mean ± SD. *p < 0.05, **p < 0.01, ***p ≤ 0.001, n = 16 retinas.

Finally, we were interested to understand how the timing of microglia depletion following low light exposure affects the expression of pro-inflammatory factors. To address this, retinal transcript levels of *Il-1β, Il-6, Tnf-α, Aif-1, Tspo* and *Ccl2* were investigated using qRT-PCR. All analyzed pro-inflammatory markers were significantly higher in retinas where microglia were first depleted and then repopulated (**Figure 8**). This implies that the replenished microglia possess a high inflammatory potential under conditions of light-damage, which may be due to the continuous presence of damage-associated signals in the degenerating retina.

## Discussion

In the present study, we provide evidence that microglia ablation with PLX3397 during light-induced retinal degeneration does not protect from photoreceptor loss. We also showed that depleting of microglia caused accumulation of cell debris and TUNEL-positive cells in the outer retina. These data contrast previous studies where beneficial effects of microglia depletion with CSF1R antagonists have been reported in the brain and retina environment. Thus, microglia depletion prevented breakdown of the blood retinal barrier, subretinal fluid accumulation, and release of pro-inflammatory cytokines in LPS induced chronic inflammation (43). CSF1R antagonist treatment also alleviated neuronal and vascular abnormalities in CX3CR1 diabetic mice (44). However, our findings are in agreement with recent studies where microglia depletion with either PLX3397 or PLX5622, caused functional impairment of rods and cones in a rat model of retinitis pigmentosa (45) and reduced visual acuity and RPE function in aged mice (46). We speculate that these differential effects are caused by different techniques and small molecules that are used to deplete microglia as well as the retinal disease models. Of note, the observation that retinal microglia were more responsive to CSF1R blockade than brain microglia may also support the idea that different effects of microglia depletion depend on the CNS compartment (28). There is also a hypothesis that microglia have region-specific phenotypes and functional states (47).

Upregulation and enhanced secretion of pro-inflammatory factors (IL-1β, IL-6, TNF-α and CCL2*)* and markers of activated microglia (AIF-1 and TSPO) occur in CNS degenerative conditions and often contribute to neuroinflammation in a positive feedback loop (48–50). In the diseased retina, activated microglia migrate into the subretinal space and enhance their secretion of cytokines and chemokines including IL-1β, IL-6, TNF-α and CCL2 (39), which contribute to macrophage recruitment and phagocytosis of photoreceptors (12). Allograft inflammatory factor 1 (AIF-1) transcripts, which encode the ionized calcium binding adapter protein 1 (Iba1), are usually upregulated by microglia in the light-damage model of AMD (36). TSPO is a highly conserved protein found in the outer mitochondrial membrane and a known marker of glial reactivity in the retina and brain (37, 51, 52). TSPO has also been associated with the development of Alzheimer’s disease and Parkinson’s disease due to dysfunctional mitochondria (53, 54). Using these markers in the present study, we conclude that PLX3397 not only depletes microglia but also impacts the microglial inflammatory repertoire and secretome.

Microglia repopulate after depletion from a resident pool of depletion resistant microglia and from peripherally invading macrophages that can develop microglia-like characteristics (55). Thus, repopulating microglia have been detected in the optic nerve, ciliary body, and central inner retina following depletion in the retina (56–58). Repopulated microglia can also secrete anti-inflammatory cytokines and trigger inhibitory signals (59). Therefore, we speculated that repopulated microglia in the lower light exposure conditions would be protective and adopt a homeostatic phenotype, promoting photoreceptor survival and proliferation. However, microglia repopulation either before or after onset of low light-induced retinal degeneration failed to restore retinal structural integrity and photoreceptor survival. We hypothesize that damage-associated signals within the degenerating environment may have primed the newly repopulated microglia to display an activated phenotype with decreased ramification and amoeboid morphology. This is in agreement with a microglia depletion study that showed that aged microglia proliferate and repopulate the brain, but these new cells still adopt a pro-inflammatory profile in the aged brain (60). Microglia repopulation studies using temporal transcriptomic analyses also revealed that microglia follow a distinct maturation program to regain their steady state (61) and potentially the time kinetics of our study may have prevented this. The newly repopulated microglia were also not able to efficiently phagocytose cell debris as we observed a higher number of TUNEL-positive cells in the lower light damage condition. We therefore suggest that at this stage of repopulation, microglia were unable to gain a similar phagocytic potential than mature phagocytes. The exact molecular mechanisms that influence this phagocytic behavior are unknown. Previously, it has been shown that CX3CR1 signaling in microglia regulates the phagocytic clearance of stressed photoreceptors (62). Thus, fluorescent beads and photoreceptor debris were phagocytosed at a faster rate and in increased numbers by *Cx3cr1*-deficient microglia and microglia recruitment into the photoreceptor layer was much higher in *Cx3cr1*-deficient *rd10* mice, which was associated with accelerated photoreceptor death and atrophy (62).

In conclusion, our study reports the retinal phenotype in conditions of light-induced retinal degeneration in the presence, absence, and repopulation of microglia. Our analyses reveal that a therapeutic usage of CSFR1-antagonists is unlikely to have beneficial effects in retinal degeneration.

## Data availability statement

The original contributions presented in the study are included in the article/**Supplementary Material**. Further inquiries can be directed to the corresponding author.

## Ethics statement

The animal study was approved by Landesamt für Natur, Umwelt und Verbraucherschutz Nordrhein-Westfalen. The study was conducted in accordance with the local legislation and institutional requirements.

## Author contributions

NL: Data curation, Investigation, Methodology, Project administration, Visualization, Writing – original draft. UK: Data curation, Investigation, Visualization, Writing – original draft, Validation, Writing – review & editing. TL: Writing – review & editing, Conceptualization, Funding acquisition, Resources, Supervision.

## Supplementary material

Supplementary Figure 1 LC-MS analysis of PLX3397 concentrations and microglia depletion in the retina. *CX3CR-1^GFP/-^* reporter mice received PLX3397 diets with 150 ppm or 1200 ppm for seven days and retinal samples were analyzed by LC-MS. **(A)** Base peak chromatogram and extracted ion chromatogram, **(B)** mass spectrum, and **(C)** PLX3397 concentrations in the retinas. **(D, E)** SD-OCT scans and heatmaps show that the two concentrations of PLX3397 have no major adverse effects on the structural integrity of the retina. **(F)** BAF imaging shows that a diet containing 150 ppm of PLX3397 was not sufficient to deplete microglia in the retina when compared to the 1200 ppm diet, which depleted almost all microglia in the retina. **(G, H)** Analysis of retinal thickness shows that a diet of 150 ppm had no effect on retinal thickness whereas a diet of 1200 ppm led to a significantly thinner retina within central and peripheral regions. Data are presented as mean ± SD. *p < 0.05, **p < 0.01, ***p ≤ 0.001, n = 9 eyes for **(C)**, and n = 4 eyes for **(G, H)**. Black scale bar = 100 μm, white scale bar = 200 μm.
