# Supplementary figures and images for: Microglia depletion/repopulation does not affect light-induced retinal degeneration in mice
Source: Front Immunol. 2024 Jan 15;14:1345382. doi: 10.3389/fimmu.2023.1345382 (PMC10822957; doi:10.3389/fimmu.2023.1345382)

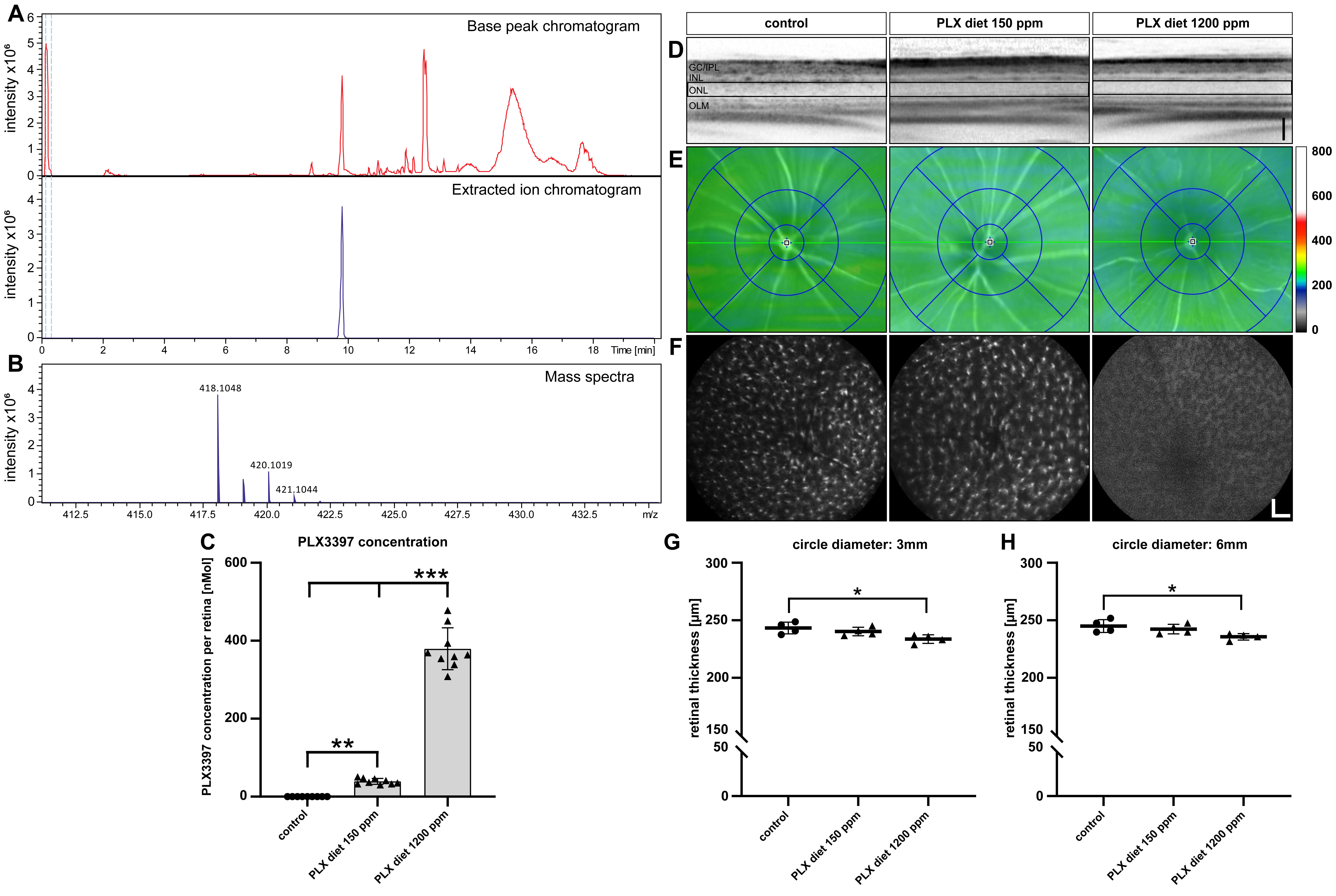

Supplement: Supplementary Figure 1 — LC-MS analysis of PLX3397 concentrations and microglia depletion in the retina. CX3CR-1GFP/- reporter mice received PLX3397 diets with 150 ppm or 1200 ppm for seven days and retinal samples were analyzed by LC-MS. (A) Base peak chromatogram and extracted ion chromatogram, (B) mass spectrum, and (C) PLX3397 concentrations in the retinas. (D, E) SD-OCT scans and heatmaps show that the two concentrations of PLX3397 have no major adverse effects on the structural integrity of the retina. (F) BAF imaging shows that a diet containing 150 ppm of PLX3397 was not sufficient to deplete microglia in the retina when compared to the 1200 ppm diet, which depleted almost all microglia in the retina. (G, H) Analysis of retinal thickness shows that a diet of 150 ppm had no effect on retinal thickness whereas a diet of 1200 ppm led to a significantly thinner retina within central and peripheral regions. Data are presented as mean ± SD. *p < 0.05, **p < 0.01, ***p ≤ 0.001, n = 9 eyes for (C), and n = 4 eyes for (G, H). Black scale bar = 100 μm, white scale bar = 200 μm. [file Image_1.jpeg]
